# Regulation of Cytochrome P450 2a5 by *Artemisia capillaris* and 6,7-Dimethylesculetin in Mouse Hepatocytes

**DOI:** 10.3389/fphar.2021.730416

**Published:** 2021-11-22

**Authors:** Sangsoo Daniel Kim, Larry Morgan, Elyse Hargreaves, Xiaoying Zhang, Zhihui Jiang, Monica Antenos, Ben Li, Gordon M. Kirby

**Affiliations:** ^1^ Department of Biomedical Sciences, Ontario Veterinary College, University of Guelph, Guelph, ON, Canada; ^2^ Chinese-German Joint Institute for Natural Product Research, College of Biological Science and Engineering, Shaanxi University of Technology, Hanzhong, China; ^3^ He’nan Joint International Research Laboratory of Veterinary Biologics Research and Application, Anyang Institute of Technology, Anyang, China

**Keywords:** Artemisia capillaris thunb, cytochrome P450, mouse, gene regulation, liver, jaundice

## Abstract

Jaundice is a potentially fatal condition resulting from elevated serum bilirubin levels. For centuries, herbal remedies containing *Artemisia capillaris* Thunb*.* including the compound 6,7-dimethylesculetin (DE) have been used in Asia to prevent and treat jaundice in neonates. DE activates an important regulator of bilirubin metabolism, the constitutive androstane receptor (CAR), and increases bilirubin clearance. In addition, murine cytochrome P450 2a5 (Cyp2a5) is known to be involved in the oxidative metabolism of bilirubin. Moreover, treatment of mice with phenobarbital, a known inducer of both CAR and Cyp2a5, increases expression of Cyp2a5 suggesting a potential relationship between CAR and Cyp2a5 expression. The aim of this study is to investigate the influence of *Artemisia capillaris* and DE on the expression and regulatory control of Cyp2a5 and the potential involvement of CAR. Treatment of mouse hepatocytes in primary culture with DE (50 μM) significant increased Cyp2a5 mRNA and protein levels. In mice, *Artemisia capillaris* and DE treatment also increased levels of hepatic Cyp2a5 protein. Luciferase reporter assays showed that CAR increases *Cyp2a5* gene transcription through a CAR response element in the *Cyp2a5* gene promoter. Moreover, DE caused nuclear translocation of CAR in primary mouse hepatocytes and increased Cyp2a5 transcription in the presence of CAR. These results identify a potential CAR-mediated mechanism by which DE regulates *Cyp2a5* gene expression and suggests that DE may enhance bilirubin clearance by increasing Cyp2a5 levels. Understanding this process could provide an opportunity for the development of novel therapies for neonatal and other forms of jaundice.

## Introduction

Jaundice is a condition that is particularly common in neonates resulting from an imbalance in the production and elimination of bilirubin (BR) that is characteristically observed during the transitional period following birth (Dennery et al., 2001; Mitra and Rennie, 2017; Chee et al., 2018). BR is produced during normal heme catabolism and displays cytoprotective capacities at physiological levels and toxicity at supra-physiological concentrations (Tomaro and Batlle, 2002; McDonagh, 2010; Abu-Bakar et al., 2011; Takeda et al., 2015). To prevent excessive accumulation of BR, hepatic uridine diphosphate 5′-glucuronosyltransferase 1A1 (UGT1A1) catalyzes the conjugation of BR to glucuronic acid to produce a water-soluble product suitable for biliary excretion. However, deficiencies in this process result in elevated serum bilirubin concentrations that is manifested clinically as jaundice (Huang et al., 2003; Mitra and Rennie, 2017; Chee et al., 2018).

Due to a more rapid turnover of erythrocytes, newborns produce bilirubin at much higher rates than adults (Mitra and Rennie, 2017; Chee et al., 2018). Additionally, infants are relatively deficient in UGT1A1, causing them to have a limited capacity to conjugate and excrete bilirubin (Yang et al., 2011; Rets et al., 2019). The primary concern and consequence of this imbalance is the ability of lipophilic bilirubin to concentrate in the central nervous system and elicit neurotoxicity, a condition called kernicterus (Mitra and Rennie, 2017). Neonatal jaundice is conventionally treated with phototherapy, a process which involves exposure of affected babies to UV light causing photoisomerization of bilirubin to an excretable form (Mitra and Rennie, 2017; Chee et al., 2018). Pharmacological therapies such as phenobarbital have been shown to enhance bilirubin excretion, however adverse effects persist (Dennery et al., 2001; Huang et al., 2003). Consequently, phototherapy remains widely used to effectively reduce bilirubin levels in infants (Mitra and Rennie, 2017).

In Asia, Yin Zhi Huang and other herbal decoctions containing *Artemisia capillaris* Thunb*.* Such as Yin Chen Hao have long been used to prevent and treat neonatal jaundice (Li et al., 2001; Li et al., 2017; Hui et al., 2020) and other liver diseases. Both Yin Zhi Huang and phenobarbital have been shown to enhance bilirubin clearance, however Yin Zhi Huang displays a more potent effect (Huang et al., 2003). The major constituent of *Artemisia capillaris* Thunb. 6,7-dimethylesculetin (DE; also known as scoparone), has been shown to accelerate bilirubin clearance in mice *in vivo* and activate an important regulator of bilirubin metabolism, the constitutive androstane receptor (CAR), in primary mouse hepatocytes (Huang et al., 2003; Elferink, 2004).

As a member of the nuclear receptor superfamily, CAR is a ligand-responsive transcription factor that is predominantly expressed in the liver (Baes et al., 1994; Pustylnyak et al., 2020). Inactive CAR is restricted to the cytoplasm where it forms a complex with heat shock protein 90 (HSP90), cytoplasmic CAR-retaining protein (CCRP), and PPP1R16A (a regulatory subunit of protein phosphatase 1β) (Savas et al., 1999; Kachaylo et al., 2011; Pustylnyak et al., 2020). Upon ligand activation, CAR is released from the protein complex to allow for nuclear translocation. Within the nucleus, CAR forms a heterodimer with the retinoid X receptor (RXR) which then binds to specific DNA-responsive elements to mediate transcriptional activation of target genes (Savas et al., 1999; Kachaylo et al., 2011). CAR is an important regulator of the expression of enzymes involved in xenobiotic metabolism including proteins involved in the metabolism and elimination of bilirubin (Huang et al., 2003; Kachaylo et al., 2011; Pustylnyak et al., 2020).

In a study conducted by Huang *et al.* (2004), treatment of humanized CAR transgenic mice with the herbal decoction Yin Zhi Huang resulted in an increased expression of functional components of the bilirubin clearance pathway and accelerated bilirubin elimination (Huang et al., 2004). This effect was absent in CAR knockout mice. Because there is evidence to suggest that several other nuclear receptors may be involved in the regulation of bilirubin clearance, Huang *et al.* (2004) compared the roles of four receptors [CAR, pregnane X receptor (PXR), aryl hydrocarbon receptor (AhR) and peroxisome proliferator-activated receptor alpha (PPARα)] (Huang et al., 2004). Following treatment with specific activators for each nuclear receptor, the most significant increase in bilirubin clearance was observed in mice treated with the CAR activator, 1, 4-bis [2-(3, 5-dichloropyridyloxy)]benzene (TCPOBOP). A more recent study has confirmed the role of CAR as an important regulator of bilirubin clearance (Wang et al., 2017).

The cytochrome P450 (CYP) enzymes comprise a superfamily of metabolically active hemoproteins. Murine Cyp2a5, and its human ortholog CYP2A6, are important hepatic enzymes involved in the metabolism of a variety of compounds including the endogenous substrate bilirubin (Seubert et al., 2002; Abu-Bakar et al., 2005; Abu-Bakar et al., 2011; Lämsä et al., 2012; Kim et al., 2013). Regulatory control of the expression of Cyp2a5 is distinct from that of other CYP enzymes as it is induced by a broad range of structurally diverse compounds and by pathophysiological states that reduce the expression of other CYPs (Abu-Bakar et al., 2007; Kirby et al., 2011; Muhsain et al., 2015). For example, hepatic Cyp2a5 is induced during liver injury caused by several hepatotoxins including phenobarbital, pyrazole, chloroform, various heavy metals and 1,4-bis [2-(3,5-dichloropyridyloxy)]benzene (TCPOBOP) (Kirby et al., 2011). Cyp2a5 expression is also upregulated by heme, bilirubin, and porphyrinogenic compounds such as aminotriazole and griseofulvin (Abu-Bakar et al., 2011; Kirby et al., 2011). Several transcription factors including the albumin D-site-binding protein (Lavery et al., 1999), the hepatocyte nuclear factor 4 (HNF-4), the nuclear factor 1 (NF-1) [5], the aryl hydrocarbon receptor (AhR) [6], and the nuclear factor (erythroid-derived 2)-like 2 (Nrf2) [2] have been implicated in the expression of Cyp2a5 (Kirby et al., 2011). CAR has also been linked to the regulation of Cyp2a5 expression. Mice treated with the CAR inducer phenobarbital displayed increased expression of Cyp2a5, an effect that was absent in CAR knockout mice (Simonsson et al., 2006; Kirby et al., 2011). Although these findings suggest that CAR may be involved in the regulation of Cyp2a5, no CAR-responsive elements have been positively identified within the promoter of the CYP enzyme.

We hypothesize that *Artemisia capillaris* Thunb*.* and DE induce Cyp2a5 expression through a transcriptional mechanism involving CAR. The aims of this study are to assess the effect of *Artemisia capillaris* and DE on Cyp2a5 expression in mouse liver and DE-treated hepatocytes in primary culture and to determine the role of CAR in transcriptional regulation of Cyp2a5 by DE.

## Materials and Methods

**FIGURE 1:**
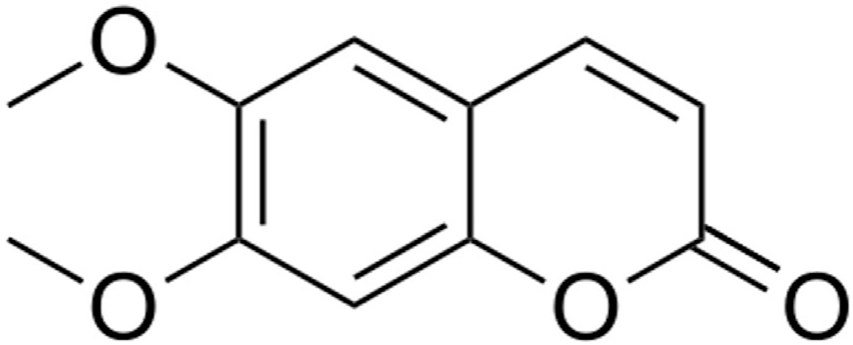
Chemical structure of 6,7-dimethylesculetin.

**Reagents**. UPLC-MS-grade acetonitrile and formic acid were purchased from Thermo Fisher Scientific (Waltham, MA, United States), Sigma-Aldrich (Darmstadt, Germany), respectively. Ultrapure water with a resistivity of 18 MΩ cm at 25°C was generated with Microporous system (Ulu pure, Xian, Shaanxi, China). The analytical standards were purchased from Yuanye Bio-Technology (Shanghai, China): 6,7-dimethylesculetin (purity >98%, MW: 206.1, CAS: 120-08-1) was purchased from Chengdu Biopurify Phytochemicals Ltd. (Chengdu, Sichuan, China). The chemical structure of 6,7-dimethylesculetin is shown in Figure 1. The plant material of *Artemisia capillaris* Thunb. originating from Shangluo, Shaanxi province, was purchased from Yikang Pharmacy (Yangling, Shaanxi, China) and was authenticated by Dr Xiaoying Zhang (Pharmacologist). A voucher specimen (No. 200701) was deposited in the Shaanxi University of Technology, Anyang, China.

**Preparation of *Artemisia capillaris* samples.** The sample of *Artemisia capillaris* was washed twice with distilled water and dried in oven at 60°C. Sample preparation for animal treatment involved preparing a decoction by boiling 40 g of *Artemisia capillaris* in distilled water for 30 min which was then filtered and adjusted to a final volume of 40 ml. Sample preparation for HPLC analysis involved extracting 5 g of dried *Artemisia capillaris* in 100 ml of 53% ethanol in water for 6 h. After filtration, ethanol was removed by evaporation, water was removed by lyophilization and the extracts were stored at −80°C for future use.

**FIGURE 2:**
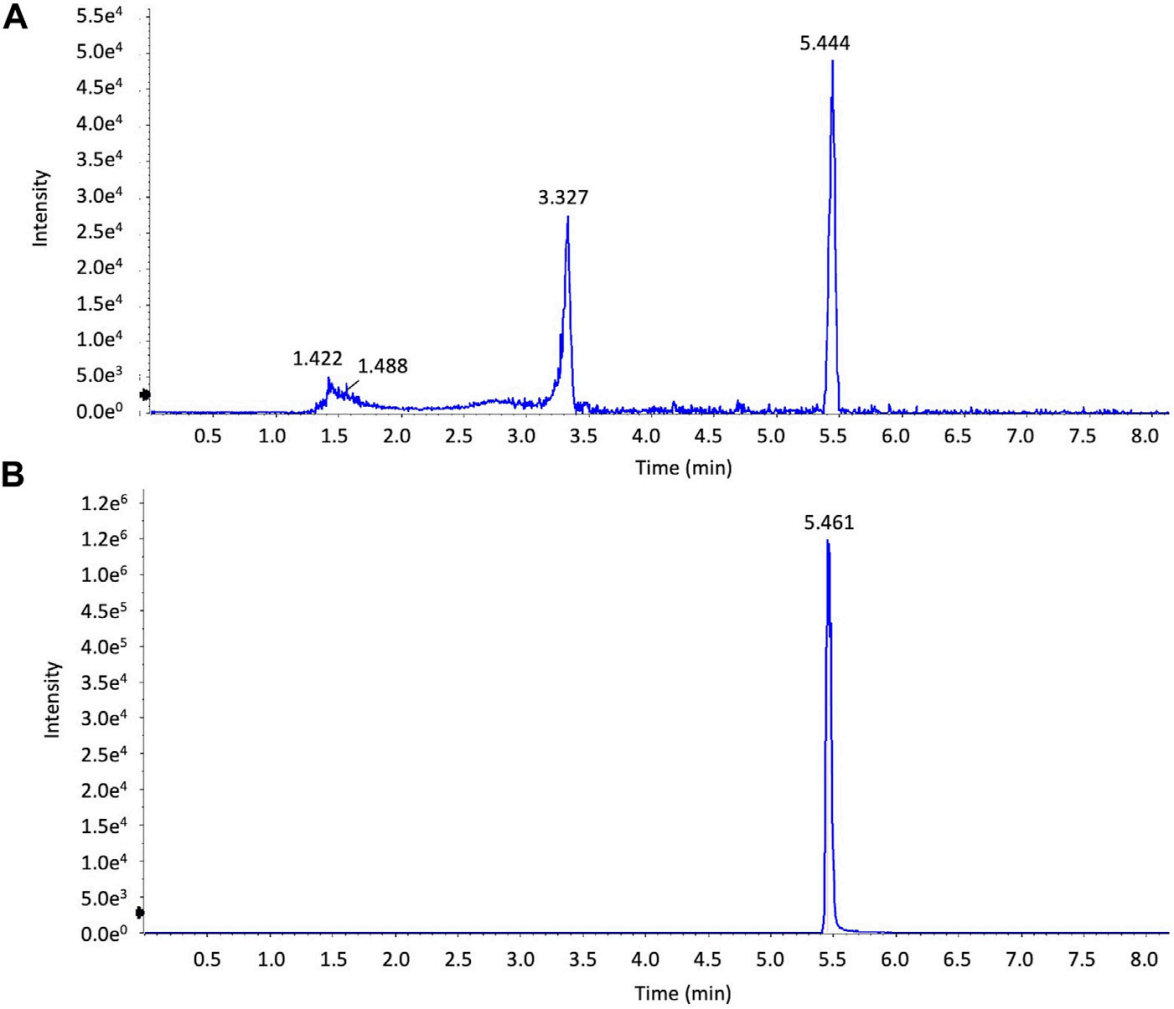
HPLC analysis of extracts of Artemisia capillaris. Extracts of *Artemisia capillaris* and the standard compound 6,7-dimethylesculetin were subjected to HPLC analysis (Panels **(A)** and **(B)** respectively).

**HPLC analysis of *Artemisia capillaris* extracts.** The various constituents in the decoction of *Artemisia capillaris* were determined by UHPLC by dissolving 5 mg of extract in 1 ml of 50% methanol and 0.1 mg of 6,7-dimethylesculetin standard in 1 ml of 50% methanol. Chromatographic separations were achieved using a Shimadzu UHPLC, LC-30 system (Shimadzu Corporation, Kyoto, Japan) and a UV-Vis photodiode array detector. Ten microliters of each dissolved extract sample was injected onto a Shimadzu InertSustain C18 liquid chromatography column (100 mm × 2.1 mm, 2 µm particles). Mobile phase A was acetonitrile and mobile phase B was ultrapure water containing 0.1% formic acid. The gradient elution program was set as follows: 5–50% (A) for 0–7 min, 50%–95% (A) for 7–9 min, 95%–95% (A) for 9–10 min. The flow rate was 1.0 ml/min. The column was maintained at 35°C. HPLC chromatograms were produced using a wavelength of 345 nm (Figure 2A,B).

**FIGURE 3:**
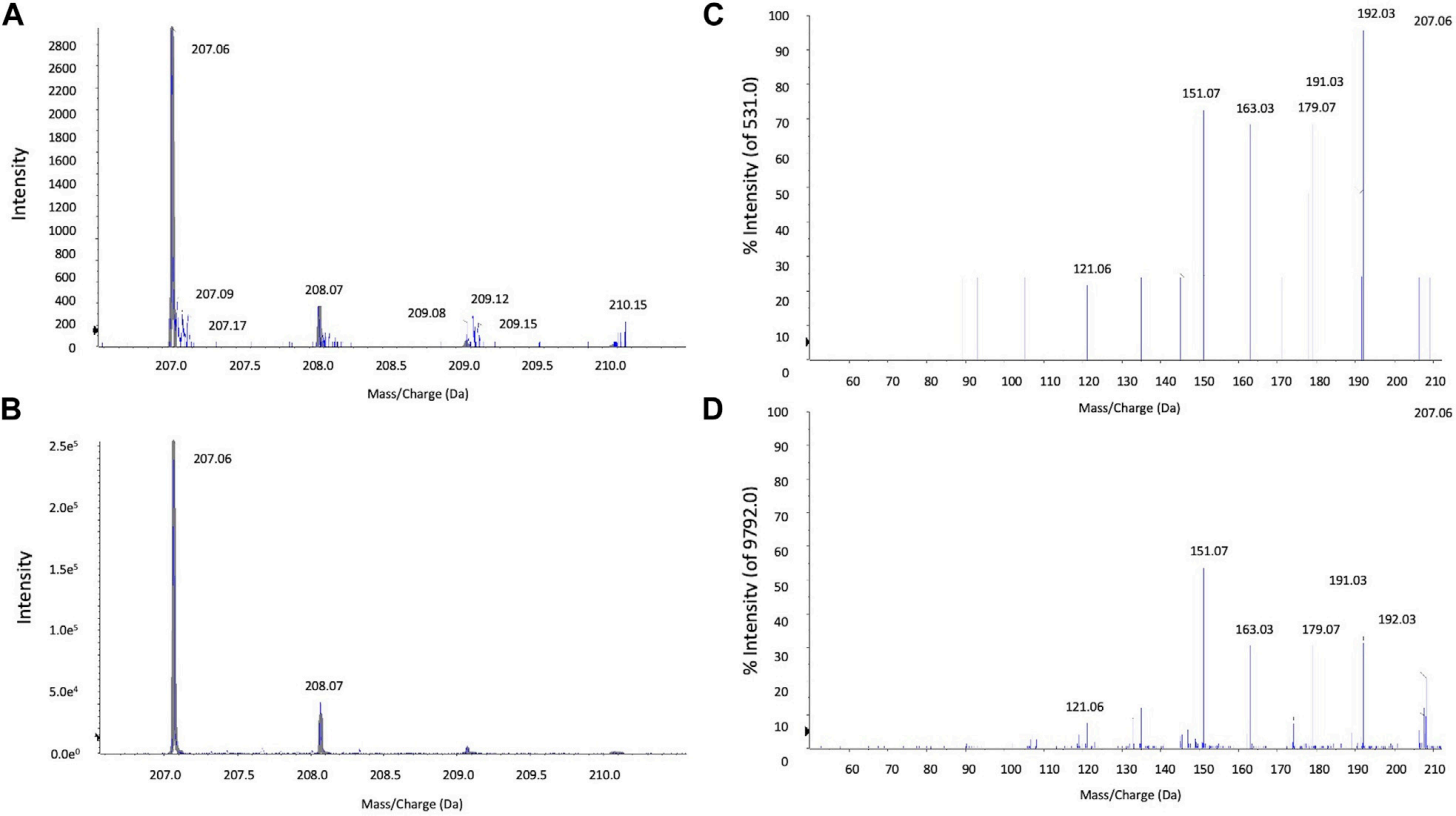
LC-MS/MS analysis of extracts of Artemisia capillaris. The major HPLC peak (5.4 min) from extracts of *Artemisia capillaris* and the standard compound 6,7-dimethylesculetin were subjected to tandem mass spectrometry. Primary isotope mass spectrometry of *Artemisia capillaris* extract and 6,7-dimethylesculetin (Panels **(A)** and **(B)** respectively) and the secondary mass spectrum of *Artemisia capillaris* extract and 6,7-dimethylesculetin (Panels **(C)** and **(D)** respectively) from precursor 207.1 Da are shown.

**Mass spectrometry of *Artemisia capillaris* extracts.** Mass spectrometry analysis of *Artemisia capillaris* extracts was achieved by Hybrid Quadrupole-TOF LC/MS/MS Mass Spectrometry using a TripleTOF^®^ 5,600 + system (Sciex, Framingham, MA). Electrospray ionization (ESI) was used to detect positive ions. The ESI source conditions were as follows: Ion Source Gas1(Gas 1):50, Ion Source Gas2(Gas 2):50, Curtain Gas (CUR): 25, Source Temperature: 500°C (positive ion), Ion Spray Voltage Floating (ISVF) 5500V (positive ion), TOF MS scan range: 100–1,200 Da, product ion scan range: 50–1,000 Da, TOF MS scan accumulation time 0.2 s, product ion scan accumulation time 0.01 s, The secondary mass spectrum was obtained by information dependent acquisition (IDA) with high sensitivity mode ±60V, Collision Energy: 35 ± 15 eV. The mass spectra of the first-order isotope (Figures 3A,B) and the secondary mass spectrum of the main fragment (Figures 3C,D) of extracts of *Artemisia capillaris* and 6,7-dimethylesculetin are presented.

**Animals.** Twelve adult male Kunming mice (20–25 g) (The Experimental Animal Center of the Medical University of the Air Force, Xi’an, China) were fed *ad libitum* and exposed to a 12-h light/dark cycle in a 23°C climate. Twelve adult male mice were divided into three groups (i.e., four controls, four *Artemisia capillaris*-treated and four DE-treated) and were gavaged daily for 3 days with either distilled water, the *Artemisia capillaris* decoction (10 ml/kg) or DE dissolved in distilled water (100 mg/kg). Mice were then euthanized by CO_2_ inhalation and liver samples were collected.

**Isolation and Culturing of Primary Mouse Hepatocytes.** Primary mouse hepatocytes were isolated from male C57Bl/6 mice using a modified two-step retrograde collagenase perfusion method as previously described (Gilmore and Kirby, 2004). Mice were first anesthetized with an intraperitoneal injection (100 mg/kg) of pentobarbital sodium (Euthanyl^®^ 240 mg/ml, Bimeda-MTC Animal Health Inc. Cambridge, ON, Canada). A catheter was inserted into the inferior vena cava and the liver was perfused with Hank’s balanced salt solution (pH 7.4), containing 0.1 M EGTA and 1 M HEPES, for 2.5 min (3 ml/min). The liver was subsequently perfused with collagenase (100 U/mL) in William’s E medium (pH 7.4), supplemented with 1 M HEPES and 7.5% (v/v) bovine serum albumin, for 5–8 min (4 ml/min) to allow for hepatocyte dissociation. The liver was excised, then rinsed and scored in fresh attachment media containing William’s E medium, pH 7.4, supplemented with 10 mM dexamethasone, ITS (5 mg/L, insulin, 5 mg/L transferrin, 5 μg/L sodium selenite), 10 μg/ml gentamicin, and 10% (v/v) fetal bovine serum. The dissociated hepatocytes were filtered, centrifuged (50 g for 2 min) and resuspended in attachment medium. Hepatocytes were plated at various densities described below and maintained in a humidified incubator (5% CO_2_, 37°C). After 8 h, the attachment medium was replaced with serum-free William’s E medium, supplemented with 1 M HEPES, ITS and 7.5 μg/ml gentamycin, and the cells were left to incubate overnight.

**Treatment of Primary Mouse Hepatocytes.** Cultured primary mouse hepatocytes were plated at a density of 9 × 10^5^ cells per well in 6-well plates and were treated with variable concentrations of DE (10, 25, 50 μM) or the vehicle dimethyl sulfoxide (DMSO, 0.1%) in serum-free William’s E medium. Hepatocytes were then maintained in a humidified incubator for 24 h for the experiment with variable doses of DE or for 1, 3, 6 or 24 h at a single dose of 50 μM.

**RNA Extraction, Reverse Transcription and Comparative Real-time RT-PCR Analysis.** Comparative real-time reverse transcription polymerase chain reaction (real time RT-PCR) was performed to assess *Cyp2a5* expression in DE-treated primary mouse hepatocytes. Following treatments described above, total cellular RNA was isolated using TRIzol^®^ reagent (Thermo Fisher Scientific, Waltham, MA, United States) as per the manufacturer’s instructions. RNA was quantified using a NanoDrop ND-1000 spectrophotometer (Thermo Fisher Scientific, Waltham, MA, United States). The RNA (1 μg) was then treated with 1 unit of DNase I (RQ1 RNase-Free DNase; Promega, Madison, WI, United States). cDNA was produced from the DNase-treated RNA by reverse transcription using 0.1 μg of random primers, 20 units of RNase inhibitor (RNase-OUT; Thermo Fisher Scientific, Waltham, MA, United States), and 200 units of Moloney murine leukemia virus reverse transcriptase (M-MLV RT; Thermo Fisher Scientific, Waltham, MA, United States). A LightCycler 2.0 apparatus (Roche Life Science, Indianapolis, IN, United States) was used to perform real-time PCR using 1 µl SYBR Green I, 2 mM Mg+ (DNA Master SYBR Green I kit; Applied Biosystems-Thermo Fisher Scientific, Waltham, MA, United States) and 5 μM of each following primers:

Cyp2a5 Forward 5′-GGA​CAA​AGA​GTT​CCT​GTC​ACT​GCT​TC-3′

Reverse 5′-GTG​TTC​CAC​TTT​CTT​GGT​TAT​GAA​GTC​C-3′

GAPDH Forward 5′-ACA​GTC​CAT​GCC​ATC​ACT​GCC-3′

Reverse 5′-GCC​TGC​TTC​ACC​ACC​TTC​TTG-3′

The PCR program parameters included an initial denaturation period (95°C for 10 min) followed by 45 amplification cycles consisting of denaturation (95°C for 15 s), annealing (70°C for 5 s), and elongation (72°C for 15 s) steps. Relative *Cyp2a5* mRNA levels of the treatment groups were determined by comparing the amplification threshold cycles at which the fluorescent signal exceeded the background level. *Cyp2a5* mRNA quantities were normalized against the housekeeping gene, glyceraldehyde-3-phosphate dehydrogenase (GAPDH), to correct for sample-to-sample variation.

**Protein Extraction and Western Blot Analysis.** Western blot analysis was performed to assess Cyp2a5 protein expression in DE-treated primary mouse hepatocytes and in DE- and *Artemisia capillaris*-treated mouse liver. Primary hepatocytes were plated at a density of 1 × 10^6^ cells per well in 12-well plates. Following a 48 h treatment with DE (5, 10, 25 and 50 μM), primary mouse hepatocytes were harvested and cell extracts were prepared in lysis buffer (50 mM Tris-HCl, pH 7.4, 250 mM sucrose, 25 mM KCl, 5 mM MgCl_2,_ and 1 Roche cOmplete Mini Protease Inhibitor Cocktail™ tablet (Sigma-Aldrich, Oakville, ON, Canada) per 10 ml as we have previously described (Nichols and Kirby, 2008). Microsomes were prepared from mouse liver by differential centrifugation according to our protocol (Kirby et al., 1993) and protein concentrations were determined by the Bradford method. Nuclear protein was isolated from primary mouse hepatocytes as we have previously described (Kim et al., 2013). Proteins from nuclear extracts (25 μg) and microsomes (50 μg) were separated by size via SDS-PAGE (4% acrylamide stacking gel, 10% acrylamide separating gel) and transferred to a nitrocellulose membrane. The membrane was blocked for 1 h at room temperature in 5% skim milk dissolved in Tris-buffered saline with 0.5% Tween-20 (TBST). Following an overnight incubation (4°C) with either chicken anti-mouse Cyp2a5 polyclonal antibody (1:10,000) (a kind gift of Dr. H. Raunio; University of Kuopio, Finland) or rabbit anti-mouse constitutive androstane receptor polyclonal antibody (ab228767, 1:2000) (Abcam, Cambridge, United Kingdom), the membrane was incubated with rabbit anti-chicken peroxidase-conjugated secondary antibody (1:5,000) (Sigma-Aldrich, Oakville, ON, Canada) for 1 h at room temperature. Chemiluminescence detection of the secondary antibody was performed by the ECL + Plus™ Western blotting method (GE Healthcare Sciences, Waltham, MA, United States) using a Typhoon 9,410 scanner (GE Healthcare Sciences, Waltham, MA, United States). The membrane was then blocked and subject to incubation with mouse anti-β-actin polyclonal antibody (1:5,000) (Sigma-Aldrich, Oakville, ON, Canada), followed by goat anti-mouse peroxidase conjugated secondary antibody (1:2000) (Sigma-Aldrich, Oakville, ON, Canada). After chemiluminescence detection, ImageJ2^®^ software was used to quantify Cyp2a5 by densitometry, relative to the housekeeping protein, β-actin (Rueden et al., 2017).

**Transfection and Dual-Luciferase Reporter Assays.** Transfections and dual-luciferase reporter assays were conducted to determine whether the DE-mediated induction of Cyp2a5 in primary mouse hepatocytes occurs via a transcriptional mechanism. First, primary mouse hepatocytes dispersed in attachment media were plated at a density of 2.5 × 10^5^ cells per well in 24-well plates. The attachment media was replaced with serum-free media 8 h later and the cells were left to incubate overnight. Hepatocytes were then transiently transfected using Lipofectamine 2000 (Thermo Fisher Scientific, Waltham, MA, United States) in Opti-MEM reduced serum media (Thermo Fisher Scientific, Waltham, MA, United States) as per the manufacturer’s instructions. All cells were transfected with the phRL-TK reporter plasmid (*Renilla reniformis*, Promega, Madison, WI, United States) (50 ng/well) to control for variability in cell numbers and transfection efficiency (Shifera and Hardin, 2010). As a positive control, hepatocytes were co-transfected with pGL3 control plasmid (Promega, Madison, WI, United States) (500 ng/well) which constitutively expresses the luciferase gene. Co-transfection with the empty pGL3 basic plasmid (Promega, Madison, WI, United States) (500 ng/well) served as a negative control. The remaining hepatocytes were co-transfected with a Cyp2a5 reporter construct (500 ng/well), which contained a 3,033 bp segment of the Cyp2a5 5′ proximal promoter [p*Cyp2a5*-3,033 + 10luc; (a kind gift of Dr. Jukka Hakkola, University of Oulu, Finland)]. To assess whether DE-induced Cyp2a5 promoter activity involves CAR, cells were also co-transfected with a murine CAR plasmid (pCR3-mCAR, 300 ng/well) kindly provided by Dr. Masahiko Negishi (NIEHS, NIH, United States)). At 24 h after transfection, the media was replaced with serum-free media supplemented with DE (50 μM) or the vehicle (DMSO). After an additional 24 h of incubation, the media was removed and the cells were lysed using a passive lysis buffer (Promega) (110 µl/well) for 1 h at −80°C. Luciferase activity was then measured with a FLUOstar OPTIMA luminometer (BMG LABTECH, Ortenberg, Germany) using a dual-luciferase reporter assay system (Promega) as per the manufacturer’s instructions. Relative luciferase activities were determined through normalization against *Renilla* luciferase activities. The data are presented as a ratio of luminescence relative to the control.

**Statistical Analysis.** Data are presented as the mean ± standard error of the mean (SEM). One-way analysis of variance (ANOVA) and Tukey’s post-hoc test were conducted to determine the statistical significance of the data generated from Cyp2a5 mRNA and protein analyses. Two-way ANOVA and Bonferroni’s post-hoc test were utilized for analysis of the luciferase assay data. *p*-values ≤ 0.05 were considered significant.

## Results

### HPLC and MS Analysis of *Artemisia capillaris* Extracts

The various constituents in the decoction of *Artemisia capillaris* were analyzed by UHPLC and mass spectrometry. Analysis of extracts of *Artemisia capillaris* by UHPLC revealed a major peak with a retention time of 5.444 (Figure 2A). A similar retention time (5.461) was obtained for the standard compound 6,7-dimethylesculetin (Figure 2B).

Mass spectrometry analysis of *Artemisia capillaris* extract revealed a main fragment of the first-order isotope with a mass-to charge ratio of 207.06 m/z for both *Artemisia capillaris* and 6,7-dimethylesculetin (Figures 3A,B). The secondary mass spectrum of the main fragment for both *Artemisia capillaris* and 6,7-dimethylesculetin had identical mass-to-charge ratios (i.e., 121.06, 151.07, 163.03, 179.07, 191.03, 192.03 and 207.06 m/z) for the most abundant ion peaks (Figures 3C,D).

### 6,7-Dimethylesculetin Increases Cyp2a5 mRNA and Protein Levels in Mouse Hepatocytes

**FIGURE 4:**
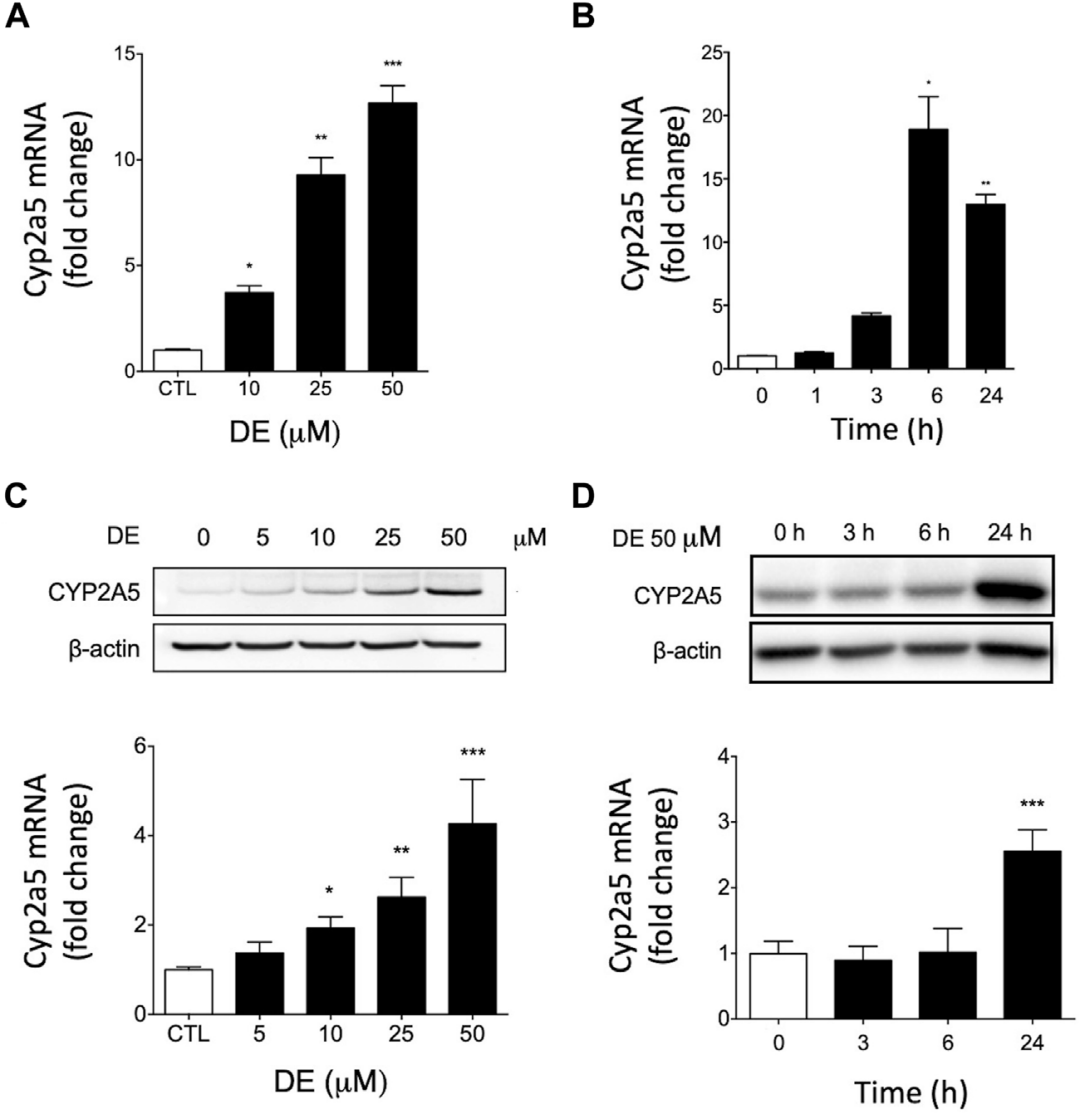
DE increases Cyp2a5 mRNA and protein levels in primary mouse hepatocytes. Primary mouse hepatocytes were treated with increasing doses of DE or the vehicle for 24 h or at 50 μM for specific times up to 24 h Cyp2a5 mRNA (Panels **(A)** and **(B)**) and protein levels (Panels **(C)** and **(D)**) were determined. Densitometric quantification of Cyp2a5 and β-Actin protein levels is presented as the mean fold-change ± SEM of the Cyp2a5/β-actin ratio relative to the control. Values represent means ± SEM of data generated from three independent experiments (n = 3) performed in triplicate. The mean difference is significant from controls at *p* ≤ 0.05 *, *p* ≤ 0.01 ** and *p* ≤ 0.001 ***.

Real-time RT-PCR of mRNA from primary mouse hepatocytes revealed dose-dependent increases in Cyp2a5 mRNA by DE to a maximum change of 12.7-fold at a concentration of 50 μM, (*p* ≤ 0.001; Figure 4A). In a time-course experiment, treatment of primary mouse hepatocytes with 50 μM DE resulted in a significant induction of Cyp2a5 mRNA that peaked at 18-fold at 6 h (*p* ≤ 0.05; Figure 4B).

Western blot analysis of microsomal protein from primary mouse hepatocytes showed that DE caused dose-dependent increases in Cyp2a5 protein levels to a maximum of approximately 4-fold at a concentration of 50 μM, (*p* ≤ 0.001; Figure 4C). In a time-course experiment, Cyp2a5 protein was significantly elevated 2.5-fold by 24 h following treatment with 50 μM DE, (*p* ≤ 0.001; Figure 4D).

### *Artemisia capillaris* Thunb. And DE Induce Murine Hepatic Cyp2a5 Protein Levels *in vivo*

**FIGURE 5:**
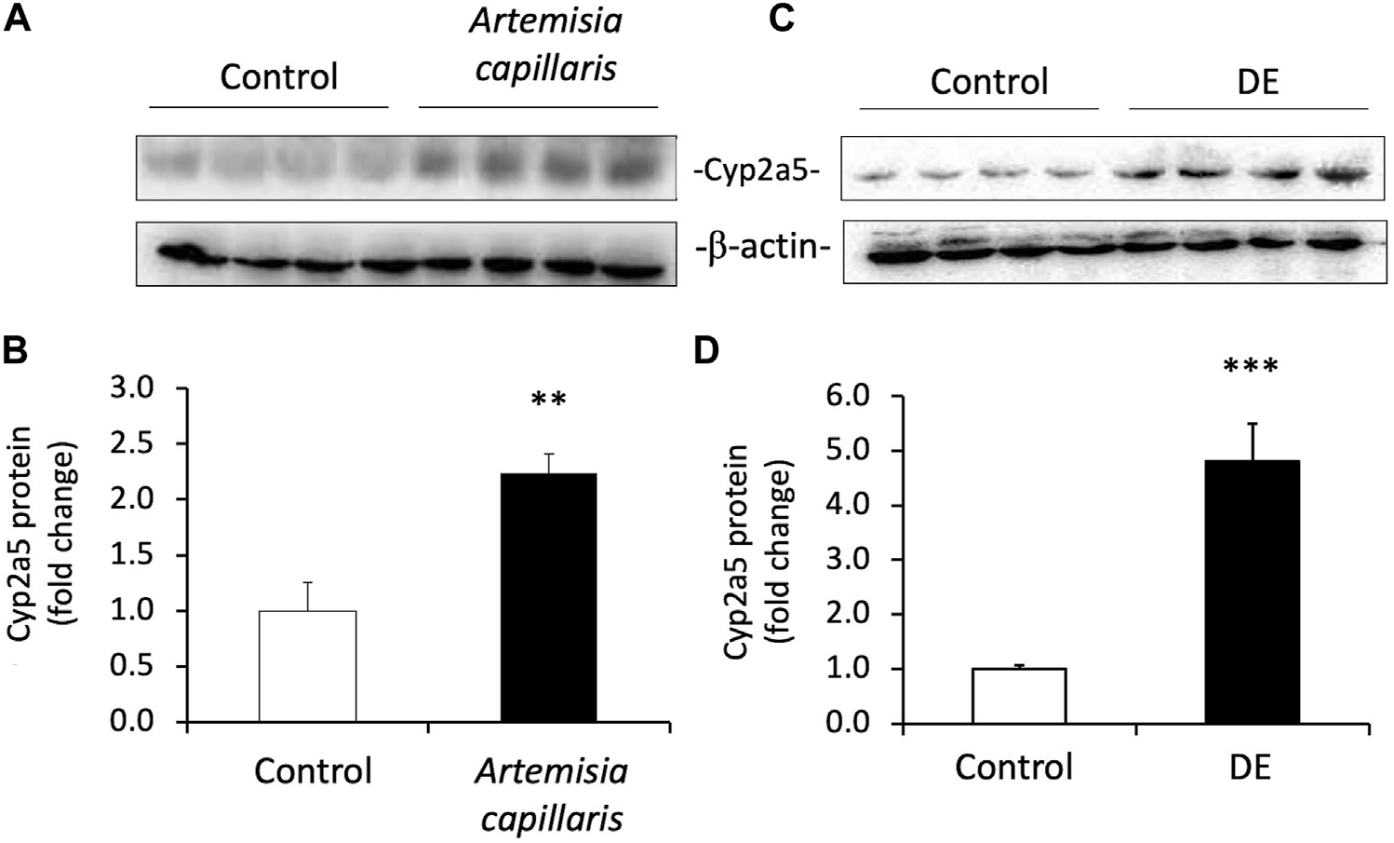
Artemisia capillaris and DE increase hepatic Cyp2a5 protein in mice**.** Groups of 4 mice were gavaged daily for 3 days with either saline (controls), *Artemisia capillaris* (10 ml/kg) or DE (100 mg/kg). Panels **(A)** and **(B)**, western blots of hepatic Cyp2a5 protein from *Artemisia capillaris*- or DE-treated mice. Panels **(C)** and **(D)**, densitometric quantification of western blots. The mean difference is significant from controls at *p* ≤ 0.01 ** and *p* ≤ 0.001 ***.

Because *Artemisia capillaris* is the active herb in Yin Chen Hao and DE is the major constituent of *Artemisia capillaris* comprising up to 2% by dry weight, we determined the capacity of both *Artemisia capillaris* and DE to induce murine Cyp2a5 protein *in vivo* (Figure 5). Both *Artemisia capillaris* and DE increased Cyp2a5 protein levels by 2.2-fold (*p* ≤ 0.01, Figure 5, Panels A and B) and 4.8-fold (*p* ≤ 0.001, Figure 5, Panels C and D) respectively.

### DE Induces Constitutive Androstane Receptor Translocation Into Nucleus

**FIGURE 6:**
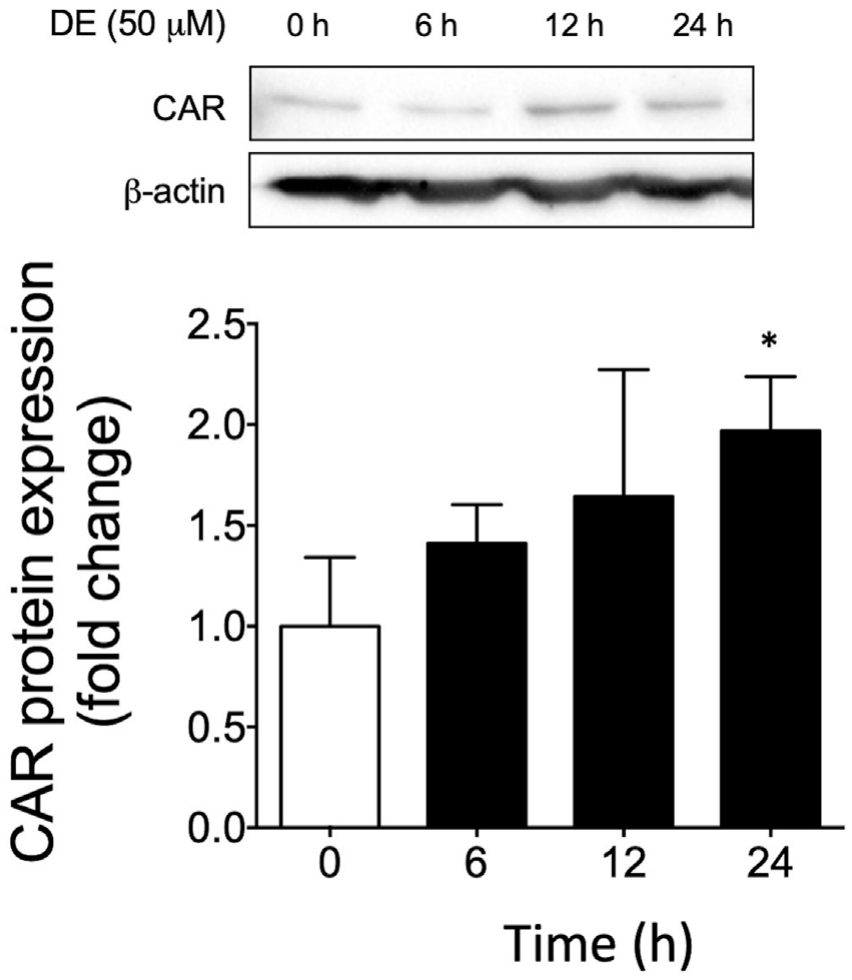
DE causes nuclear translocation of CAR protein. Primary mouse hepatocytes were treated with 50 μM DE for 0, 6, 12, and 24 h. **(A)** Western blot analysis of nuclear CAR protein from DE-treated primary mouse hepatocytes. β-actin protein levels are shown as a control for protein loading. The western blot represents one replicate of three samples. All values represent the mean ± SEM (n = 3 hepatocyte cultures from different mice) normalized against the control levels and quantified by densitometry. Mean difference is significant from the control group at * *p* ≤ 0.05.

To ascertain whether CAR is involved in DE-mediated upregulation of Cyp2a5, we first determined if DE activates CAR translocation to the nucleus. Western blot analysis of nuclear extracts from primary mouse hepatocytes demonstrated that DE treatment caused progressive increases in nuclear CAR levels in a time-dependent manner to a level significantly higher than controls by 2-fold (*p* ≤ 0.05) by 24 h (Figure 6).

### Constitutive Androstane Receptor Regulates *Cyp2a5* Transcription Through a CAR Response Element in the *Cyp2a5* Promoter

**FIGURE 7:**
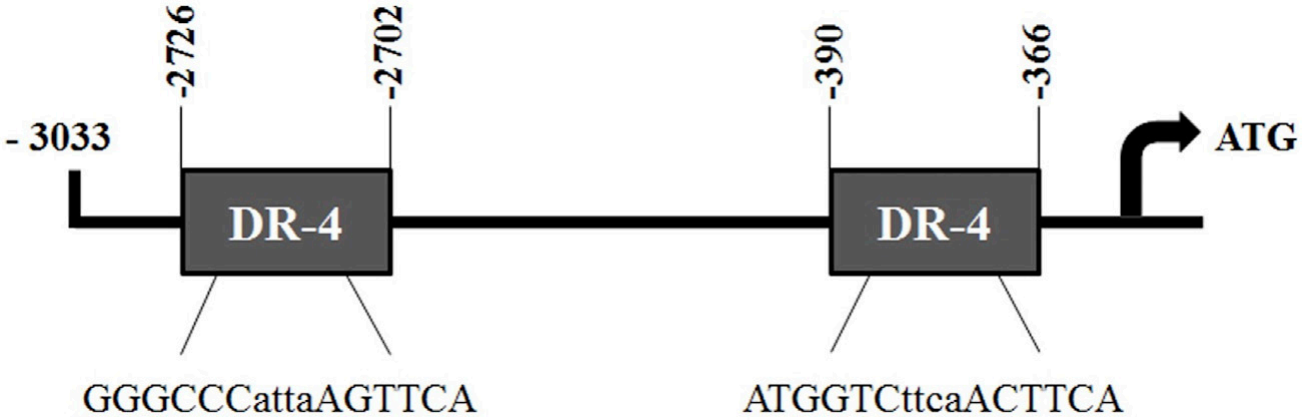
Putative CAR responsive DR-4 elements in the Cyp2a5-3,033 + 10-luc promoter construct. Two potential CAR-responsive DR-4 elements identified within the Cyp2a5-3,033 + 10-luc construct through MatInspector analysis. Core sequences are shown below each site.

**FIGURE 8:**
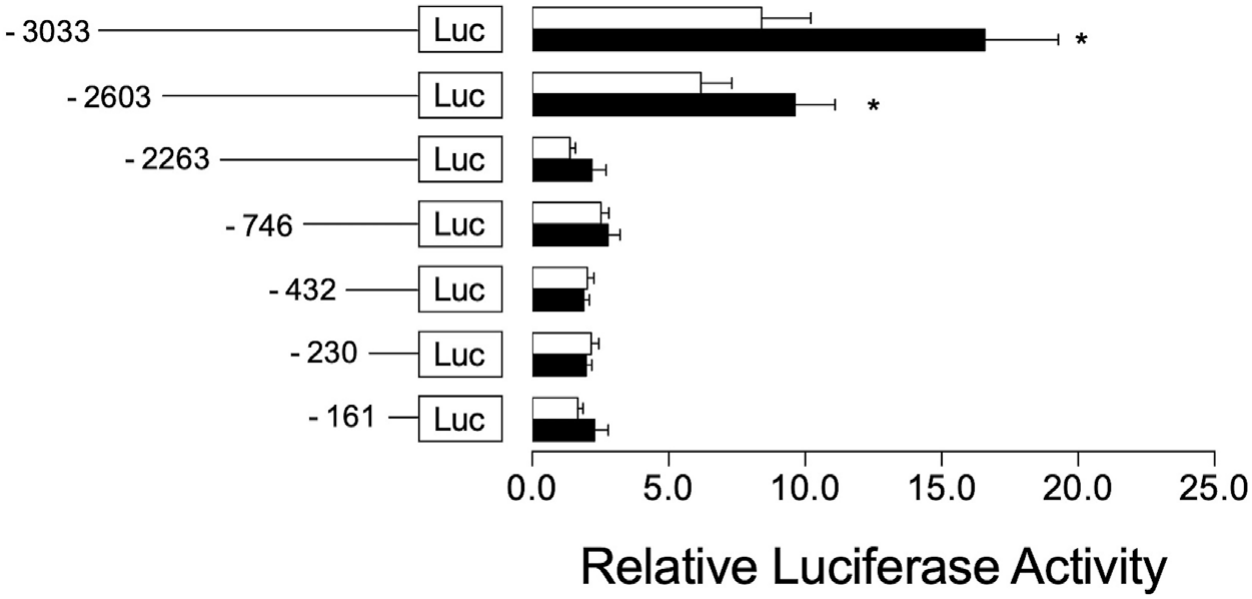
CAR co-transfection increases Cyp2a5 transcriptional activation. Mouse Cyp2a5 promoter reporter assays were conducted in primary mouse hepatocytes transfected with progressively truncated luciferase reporter constructs (white bars) and co-transfected with a CAR expression plasmid (black bars). Luciferase activities were measured 24 h after transfection. The measured activities were normalized against Renilla (pR-TK) activities. All values represent the mean ± SEM derived from n = 3 hepatocyte cultures from different mice. The effect of CAR on each reporter construct is indicated by fold activity relative to control hepatocytes co-transfected with pcDNA3.1. Mean difference is significant from the control group at *p ≤ 0.05.

To investigate the regulatory mechanism by which Cyp2a5 is induced by *Artemisia capillaris* and DE and the involvement of CAR in this process, we first identified two potential CAR-responsive DR-4 elements within the 5′-UTR of the *Cyp2a5* gene through MatInspector analysis, one located proximally within positions -366 to -390, and the other distally within positions 2,702 to 2,726 (Figure 7). To localize the CAR response element, luciferase activity was measured in mouse hepatocytes co-transfected with the mCAR expression plasmid and various truncation constructs of the *Cyp2a5* promoter (Figure 8). CAR overexpression significantly induced luciferase activity in the reporter constructs containing −3,033/+10 and −2,603/+10 fragments by approximately 16.5-fold and 10-fold respectively (*p* ≤ 0.05) but had no effect on the constructs of shorter length.

**FIGURE 9:**
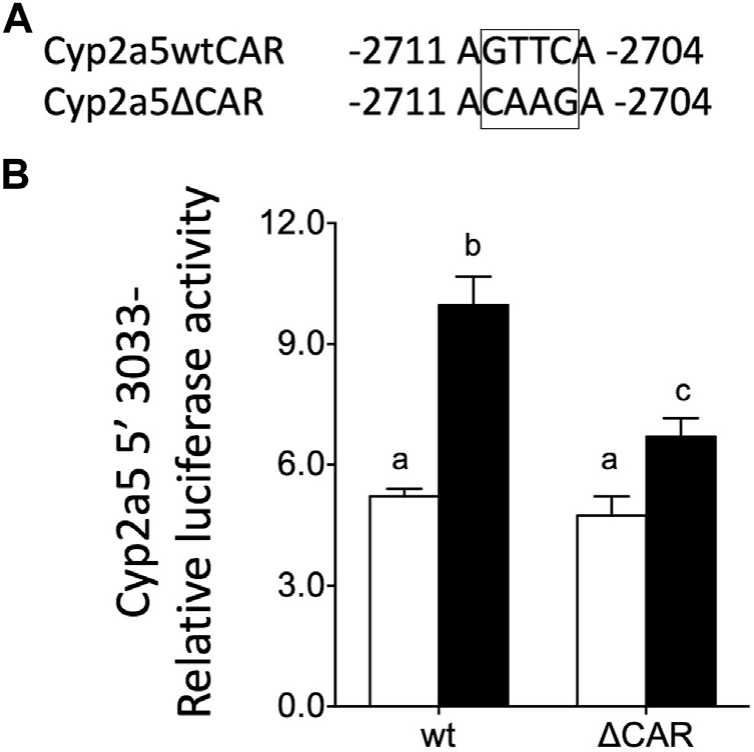
Murine CAR overexpression increases Cyp2a5 transcription via a CAR response element in the murine Cyp2a5 promoter region. **(A)** The sequence of a CAR response element within the *Cyp2a5* promoter region showing site-directed mutation. **(B)** Cyp2a5-5′-3,033 luciferase reporter activities were assessed in primary mouse hepatocytes co-transfected with a CAR expression plasmid (black bars) or without co-transfection (white bars). In addition, the effect of site-directed mutation of the CAR response element (ΔCAR) on Cyp2a5 transcriptional activation of Cyp2a5 was tested. The measured activities were normalized against Renilla (pRL-TK) activities. All values represent the mean ± SEM (n = 4 hepatocyte cultures from different mice). Bars with different letters are significantly different (*p* ≤ 0.05).

To determine the role of the distal CAR-responsive element in Cyp2a5 transactivation, we transfected primary mouse hepatocytes with the wild-type full-length luciferase reporter plasmid p*Cyp2a5*-3,033 + 10-luc or with a reporter construct with the full-length 5′UTR in which the CAR-responsive element was mutated i.e. p*Cyp2a5*-ΔCAR (Figure 9A). Co-transfection with a mCAR expression plasmid revealed that CAR overexpression increased the wild-type Cyp2a5 promoter activity two-fold (*p* ≤ 0.05), compared to hepatocytes transfected with an empty vector (Figure 9B). However, mutation of the CAR response element significantly reduced the CAR-mediated increase in reporter activity to 30% of the level observed in wild-type transfectants (*p* ≤ 0.05).

### Dimethylesculetin Increases *Cyp2a5* Transcription in the Presence of Constitutive Androstane Receptor

**FIGURE 10:**
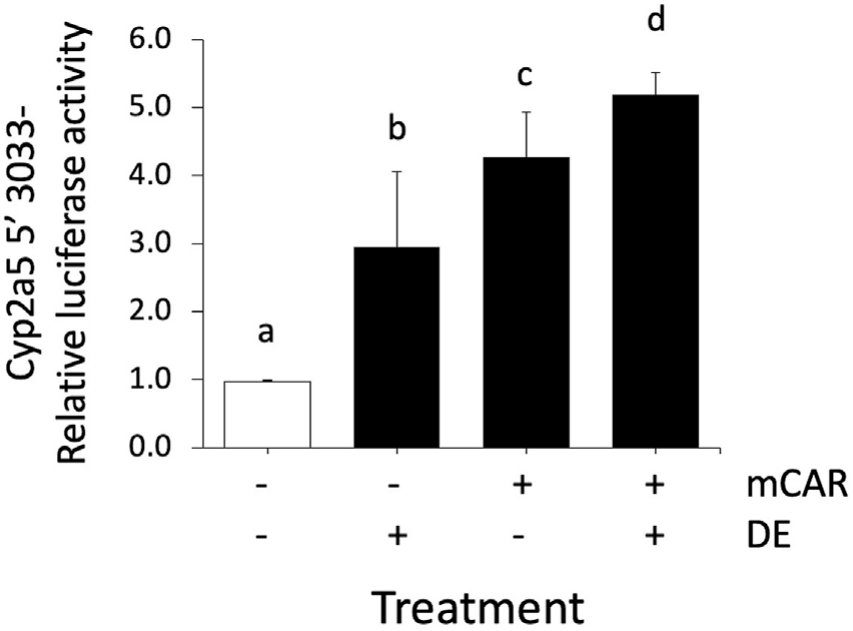
Cyp2a5 induction by DE is increased by CAR overexpression. Cyp2a5-5′-luciferase reporter activities were assessed in primary mouse hepatocytes 24 h after CAR co-transfection, CAR co-transfection followed by 50 μM DE or DE treatment alone for 24 h. The measured activities were normalized against Renilla (pRL-TK) activities. All values represent the mean ± SEM (n = 4). Bars with different letters are significantly different (*p* ≤ 0.05).

To further investigate the mechanism by which DE increases Cyp2a5 mRNA levels through a transcriptional mechanism involving CAR, a luciferase reporter assay was performed using primary mouse hepatocytes transfected with the wild-type *Cyp2a5*-3,033 + 10-luc construct with or without mCAR co-transfection in the presence or absence of DE (50 µM). A 3-fold increase in Cyp2a5 promoter activity (*p* ≤ 0.05) was observed following treatment with DE (50 µM) alone (Figure 10). Cyp2a5 promoter activity increased 4.2-fold (*p* ≤ 0.05) in hepatocytes co-transfected with mCAR and a 5-fold increase (*p* ≤ 0.05) in Cyp2a5 promoter activity was observed in DE-treated hepatocytes co-transfected with mCAR.

## Discussion

Herbal decoctions such as Yin Zhi Huang and Yin Chen Hao containing *Artemisia capillaris* Thunb. and essential constituent 6,7-dimethylesculetin (DE) have been used for centuries in Asia to prevent and treat neonatal jaundice (Hui et al., 2020). While Yin Zhi Huang increases clearance and elimination of bilirubin via a process involving the transcription factor CAR and induction of hepatic glucuronosyl transferase (Huang et al., 2003; Huang et al., 2004), the detailed mechanism underlying this therapeutic effect is not entirely clear. CAR and other xenobiotic nuclear receptors are key intermediators by which xenobiotics regulate the expression of enzyme and transporters involved in their own absorption, metabolism and eventual elimination (Yan and Xie, 2016; Negishi et al., 2020). Moreover, the elimination of potentially toxic endogenous substances, such as bilirubin, is facilitated by CAR. For example, stimulation of CAR increases expression of hepatic organic anion transporting polypeptides (OATP) 1A1 and 1A4 thereby increasing uptake of bilirubin by the liver and also induces hepatic UDP-glucuronosyltransferase (UGT) 1A1, the only transferase capable of conjugating bilirubin to increase its hydrophilicity and excretion (Wagner et al., 2005; Wang et al., 2017; Weber et al., 2021). Accordingly, exposure to DE stimulates CAR in primary mouse hepatocytes and enhances bilirubin clearance (Huang et al., 2003; Huang et al., 2004). Because CAR stimulation induces Cyp2a5 (Kirby et al., 2011), an enzyme involved in the oxidative metabolism of bilirubin (Abu-Bakar et al., 2005; Kirby et al., 2011; Kim et al., 2013), we hypothesized that DE and *Artemisia capillaris* cause overexpression of Cyp2a5 via a molecular mechanism involving transcriptional activation by CAR.

To test this hypothesis, Cyp2a5 mRNA and protein levels were first measured in *Artemisia capillaris*- and DE-treated mouse liver and DE-treated hepatocytes using quantitative real-time polymerase chain reaction (qRT-PCR) and Western blot analysis. Cyp2a5-luciferase reporter assays were then performed to investigate the involvement of CAR in the DE-mediated regulation of Cyp2a5 expression. The dose of DE (100 mg/kg) administered to mice was chosen based on the study by Huang et al., 2004 that demonstrated CAR activation and increased bilirubin clearance in mice treated with i. p. injections of DE at a dose of 100 mg/kg (Huang et al., 2004). An approximation of the human equivalent dose (HED) of DE can be derived by allometric scaling from mice to humans (HED = 100 mg/kg X 0.08 = 8 mg/kg) (USFDA et al., 2005; Nair and Jacob, 2016). The *in vitro* concentration of DE (50 μM) was also derived from the same study (Huang et al., 2004) that showed increased Cyp2b10 mRNA levels in cultured hepatocytes from wild-type mice but not from CAR knockout mice. An approximate extrapolation of this concentration of DE used in our cultured mouse hepatocyte experiments revealed it to be 14-fold lower than the HED.

Our findings show a dose-dependent relationship between DE treatment and increased Cyp2a5 expression at the mRNA and protein levels in primary mouse hepatocytes. An increase in hepatic Cyp2a5 protein levels was also observed in mice treated with both *Artemisia capillaris* and DE *in vivo*. A recent study using mouse and human liver microsomes has shown that both Cyp2a5 and CYP2A6 contribute to the O-demethylation of DE to scopoletin, the primary route of DE metabolism (Fayyaz et al., 2018). Collectively, this suggests that DE-mediated induction of Cyp2a5 would expedite DE metabolism as well as that of bilirubin, however, this was not investigated in the current study.

To determine whether DE regulates *Cyp2a5* at the transcriptional level, a luciferase reporter assay was conducted in DE-treated primary hepatocytes co-transfected with the Cyp2a5-3,033 + 10-luc promoter and the mCAR expression plasmid. DE significantly increased *Cyp2a5* promoter activity in mouse hepatocytes that overexpressed CAR indicating that DE transactivation of *Cyp2a5* occurs through a transcriptional mechanism involving CAR. We also confirmed previous studies that demonstrate DE-mediated translocation of CAR to the nucleus of mouse hepatocytes in primary culture (Huang et al., 2004; Yang et al., 2011). Within the nucleus, CAR-RXR heterodimers interact with *cis-*elements in CAR-regulated genes. Although no CAR-responsive elements have been positively identified within the Cyp2a5 gene, analysis of the Cyp2a5 5′-UTR sequence using a transcription factor binding site search (MatInspector, Genomatix), revealed two putative CAR-responsive elements. Both identified sites were direct repeat elements separated by 4 base pairs (DR-4). Our luciferase reporter results including deletion analysis and site-directed mutagenesis of these sites indicate that the more distal site represents a DR-4 motif by which CAR regulates Cyp2a5 transcriptional activity. To the best of our knowledge, this is the first time that a functional CAR-responsive DR-4 motif has been identified in the promoter region of Cyp2a5. It has previously been shown that phenobarbital causes CAR-RXR heterodimers to bind to DR-4 motifs resulting in up-regulation of CYP2B genes and other genes involved drug metabolism (Kakizaki et al., 2003). CAR also interacts with DR-4 motifs in the promoters of several other CYP genes including the murine CYP2B10 (Honkakoski et al., 1998), CYP2C29 (Ferguson et al., 2005), and CYP2C37 (Jackson et al., 2006), and human CYP2B6 (Sueyoshi et al., 1999), CYP2C9 (Gerbal-Chaloin et al., 2002), CYP2C19 (Chen et al., 2003), and CYP2C8 (Ferguson et al., 2005). These previous findings corroborate the results of this study as they highlight the potential for CAR-mediated regulation of the *Cyp2a5* gene*.*

It is not surprising that DE and *Artemisia capillaris* induce Cyp2a5 via CAR considering that CAR is xenobiotic sensor and Cyp2a5 plays an important cytoprotective role in protecting against liver injury and hepatoxicity (Kirby et al., 2011; Abu-Bakar et al., 2013; Hong et al., 2016). CAR is also involved in enhancing CYP2B10-mediated detoxification of ethanol in the liver (Cederbaum, 2012). In addition to CAR, *Cyp2a5* is regulated by other transcription factors including Nrf2 (Abu-Bakar et al., 2007; Lämsä et al., 2010; Kirby et al., 2011), AhR (Arpiainen et al., 2005) and hepatic nuclear factor 4 alpha (NRF-4a) and NF-1 (Ulvila et al., 2004) and functional response elements for these transcription factors have been identified in the *Cyp2a5* promoter region. In addition, we have also shown that bilirubin causes Nrf2-mediated transactivation of *Cyp2a5* thus providing protection against bilirubin hepatotoxicity (Kim et al., 2013).

CYPs are known to be induced and inhibited by various drugs, herbal remedies, and toxic compounds (Hakkola et al., 2020). Interestingly, Artemisinin, an extract from the plant *Artemisia annua,* used as a medication to treat malaria, is also an activator of CAR and an inducer of Cyp2b10 and Cyp2a5 in mouse liver (Simonsson et al., 2006). Moreover, human CYP2A6 is induced by various natural products including genistein and quercetin (Hakkola et al., 2020). While CYP2A6 is also induced by CAR and other transcription factors including, PXR, ERα, NRF2, HNF-4alpha, C/EBPalpha and beta, and Oct-1 (Pitarque et al., 2005; Raunio and Rahnasto-Rilla, 2012), it not is not known whether DE or *Artemisia capillaris* are capable of inducing CYP2A6. However, coumarin is the marker substrate for human CYP2A6 and mouse Cyp2a5 as they both have coumarin 7-hydroxylase activity (Kaipainen et al., 1984; Pelkonen et al., 2000; Raunio and Rahnasto-Rilla, 2012). Interestingly, DE and esculetin (6,7 dihydroxycoumarin) are both coumarin derivatives and have antioxidative and cytoprotective properties against hepatotoxicity and liver injury (Atmaca et al., 2011). It is possible that the hepatoprotective qualities of Yin Zhi Huang and Yin Chen Hao in humans and other species are due, in part, to induction of CYP2A enzymes.

In conclusion, the results of this study indicate that *Artemisia capillaris* and DE induce Cyp2a5 expression at both mRNA and protein levels. Additionally, the current findings show that 6,7-dimethylesculetin significantly increases *Cyp2a5* expression at the transcriptional level through transactivation by CAR. These findings contribute to our understanding of Cyp2a5 regulation and may also provide further insight into the mechanism by which DE enhances bilirubin clearance. While it has been well established that CAR increases bilirubin clearance by inducing hepatic UGT1A1 and various ion transporters that increase bilirubin clearance, it is possible that DE-mediated upregulation of Cyp2a5 may also be involved in view of the role of Cyp2a5 in the oxidative metabolism of bilirubin (Abu-Bakar et al., 2011; Kim et al., 2013). Future *in vivo* studies could assess the role of Cyp2a5 and CYP2A6 in the oxidative metabolism of bilirubin in DE-mediated bilirubin clearance. This may ultimately provide an opportunity for the development of novel therapies for neonatal and other forms of jaundice.

## Data Availability

The raw data supporting the conclusions of this article will be made available by the authors, without undue reservation.

## Abbreviations

AhR, aryl hydrocarbon receptor; CAR, constitutive androstane receptor; CCRP, cytoplasmic CAR-retaining protein; C/EBP, CCAAT/enhancer-binding protein; CYP, cytochrome P450; Cyp2a5, murine cytochrome P450 2a5; DE, dimethylesculetin; DR-4, direct repeat elements separated by 4 base pairs; ERα, Estrogen receptor alpha; HNF-4, hepatocyte nuclear factor 4; HSP90, heat shock protein 90; NF-1, nuclear factor 1; NRF-4α, nuclear factor 4 alpha; Nrf2, nuclear factor (erythroid-derived 2)-like 2; OATP, organic anion transporting polypeptides; PPARα, peroxisome proliferator-activated receptor alpha; PPP1R16A (a regulatory subunit of protein phosphatase 1β); PXR, pregnane X receptor; RXR, retinoid X receptor; TCPOBOP, 1, 4-bis [2-(3, 5-dichloropyridyloxy)]benzene; TBST, Tris-buffered saline with 0.5% Tween-20; UGT1A1, uridine diphosphate 5′-glucuronosyltransferase.
